# A quantitative evaluation of the use of medical lasers in German hospitals

**DOI:** 10.1002/jbio.201900238

**Published:** 2019-12-11

**Authors:** Moritz Späth, Florian Klämpfl, Florian Stelzle, Martin Hohmann, Benjamin Lengenfelder, Michael Schmidt

**Affiliations:** ^1^ Institute of Photonic Technologies Friedrich‐Alexander‐Universität Erlangen‐Nürnberg Erlangen Germany; ^2^ Erlangen Graduate School in Advanced Optical Technologies Erlangen Germany; ^3^ Department of Oral and Maxillofacial Surgery University Hospital Erlangen Erlangen Germany

**Keywords:** hospitals, laser procedures, laser surgery, medical laser

## Abstract

The laser has become an integral part of modern medicine, procedures based on this technique have found their way into a multitude of medical disciplines. There is, however, no data available on the detailed quantitative development of laser use in the medical sector. This fact gave rise to the idea of the present study, which analyzed the raw data of the quality report of German hospitals with respect to this subject. Over the 9 years of report, a steady increase in the cumulative number of cases was evident, although not all body regions in which the medical laser is used followed this trend. The CO_2_ laser was found to be the most commonly applied laser, even though a large spectrum of different laser types is used. Based on the present study, the importance of the laser for medical purposes can be confirmed.
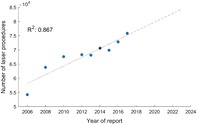

## Graphical abstract

The laser has become an integral part of modern medicine. To gain knowledge about the detailed quantitative development of laser use for medical interventions in hospitals, the present study analyses the raw data of the quality report of German hospitals. Over the 9 years of report, a steady increase in the cumulative number of cases is evident. The present study is able to confirm and substantiate the importance of the laser for medical purposes.

## 1 INTRODUCTION

One of the many scopes of application of laser systems is the medical sector. It is not only the flexibility in the application and the parameters, both with regard to the available wavelengths and the operating mode (continuous wave vs pulsed mode) that have helped the laser to be the subject of numerous past and current research projects. The different possible wavelengths allow the processing of a variety of tissue types 1, 2 and make it possible to achieve different optical penetration depths. This provides the possibility to perform medical procedures at different treatment depths—prominent examples are the removal of tattoos 3, varicose veins 4, port‐wine stains 5 and posterior capsular opacification (“after‐cataract”) 6 as well as the coagulation of vessels in the depth 7, 8. Supplementary, laser pulses up to the fs range enable high‐precision treatments 9. Since such systems have found their way into hospitals and doctors' offices, they have taken on a role in today's medicine that is no longer negligible in terms of both diagnosis and therapy 10, 11.

Looking at the various medical disciplines, there is a procedure in almost every discipline that includes the use of a laser, even if it is only the relatively simple procedure of laser coagulation, for instance. In particular, the dental 9, 12, 13, cardiovascular 9, 14, 15, urologic (urologic stone fragmentation 16, prostate vaporization/enucleation 17, 18) and most importantly the ophthalmologic areas 2, 9, 19, 20, 21 should be mentioned here. Also the wide fields of skin ablation 22, oral surgery 23 and cancer treatment are of significance, the latter particularly in urology, gynecology, neurosurgery and pulmonology 10, 24, 25.

The success story of the use of lasers in medicine could not be reduced by investigations on the topic of laser‐generated air contaminants: Pierce et al. reviewed articles on that topic leading to the conclusion that health personnel are exposed to about 150 chemical constituents of the laser‐induced plume, among them fine and ultrafine particles. They pointed out that present control mechanisms might not offer sufficient protection against this 26.

A further concern toward the use of lasers in the medical field is the fact that this technology involves a risk of inadvertently lasering structures other than those actually intended 27. Even though feedback mechanisms are already being researched, this is still an open issue, for example in laser surgery 28, 29. Furthermore, safety precautions such as the use of protective goggles, the nomination of a safety officer, the equipment of doors with appropriate protective circuits and the training of the entire operating room staff with laser safety rules must be applied in order not to endanger the patient, the operator or other present persons such as nurses 30, 31. Not least, such laser systems are expensive and therefore associated with high investments 27.

Considering the large number of scientific articles on the use of lasers in medical procedures that have been published in recent years and decades—both those that report advantages and those that report drawbacks—, the question arises in what quantity laser systems are nowadays used in day‐to‐day clinical routine. As there are currently no scientific studies on this subject, neither with regard to an absolute number of laser interventions (per time unit) nor with regard to details such as the quantitative distribution of the laser types used, the aim of this publication is to quantify the use of lasers as a tool to support medical procedures.

## 2 MATERIALS AND METHODS

The raw data underlying this publication is taken from the quality reports of German hospitals (“Qualitätsberichte”) according to § 137 Abs. 3 Satz 1 Nr. 4 SGB V, a law that obliges German hospitals to submit their data. The data analysis included data for the years of report 2006, 2008, 2010 as well as for the years 2012 up to 2017 annually; this corresponds to all quality reports available up to now, all subsequent data deliveries (“Nachlieferungen”) were included.

In addition to a multitude of other information, these reports contain quantitative data on procedures performed at each individual German hospital. The procedures are encoded according to the so‐called “Operationen‐ und Prozedurenschlüssel (OPS)” catalog, which is derived from the International Classification of Procedures in Medicine system 32. The coding of the procedures in this format allowed to specifically look at such procedures in which a laser is involved. Using MATLAB R2018b, a total of 16 153 xml files from a total of 2187 different hospitals (different sites of the same clinic were considered as one clinic) were evaluated, spread over the years of report as shown in Table 1.

**Table 1:** Number of hospitals per year in total and with laser procedures in the specific year of report

Year of report	Number of hospitals in total	Number of hospitals with laser procedures
2006	1940	364
2008	1922	432
2010	1872	440
2012	1737	489
2013	1746	491
2014	1757	499
2015	1742	504
2016	1722	480
2017	1715	509

In order to make the raw data of the single xml files accessible, a MATLAB script was created. This script centers on creating a variable with the following three dimensions: institutional identifier (“Institutionskennzeichen”), OPS code, year of report. Thereby, the institutional identifier is a nine‐digit number used to assign the data to the respective hospital. After running the script, this variable contains all available information and serves as database for the analyses of the present paper; it is of the size 2187 × 39 188 × 9.

A procedure was considered a laser procedure and thus included in the evaluation if it had the keyword “laser” in its title. This applied to 240 procedures. After identifying these laser procedures, the corresponding numbers of cases for all hospitals and all years of report were extracted from the variable mentioned before, again using a MATLAB script. The OPS codes of these 240 procedures can be found listed in the Appendix of this publication. A total of 822 different hospitals were performing at least four laser procedures in at least one of the years of report; the corresponding annual values can as well be found in Table 1. Note that if less than four people have been treated with a certain procedure, the number of cases is not stated in the quality reports, which is necessary due to data protection requirements.

The same approach was used for the analysis of the laser types. Eleven different categories for the different laser types are coded in the OPS system. For further analysis, this data was extracted from the variable mentioned before, too. Again, in the Appendix of this publication, the corresponding OPS codes can be found.

Techniques such as photodynamic therapy (PDT), for which either lasers or broadband radiators and LEDs can be used as light source and which therefore cannot be clearly attributed to the laser procedures, were not included in the analyses. For completeness, however, numbers of cases on PDT can be found in the Appendix.

Since the OPS coding of the performed procedures serves as the basis for the accounting of the hospitals with the health insurances, it can be assumed that only a negligibly small proportion of procedures were not OPS coded. At the same time, this also means that all newly approved procedures are constantly integrated into the coding. This in turn ensures that the quality reports are continually incorporating the latest medical procedures and interventions.

In the following results section, this data are presented descriptively as well as analyzed statistically using linear regressions. A linear regression analysis is a statistical technique to examine the relationship between an independent and a dependent variable. Thereby, the dependent variable is described by a linear function of the independent variable. A regression line is fitted to the data so that the sum of the squares of all residuals is minimal. Intercept and slope are estimated, characterizing this regression line 33, 34.

In the present paper, the year of report is set as the independent variable (ie, as the only predictor *x*_1_) of all conducted linear regressions, while the dependent variable is the number of laser procedures examined in the respective section (ie, the output *Y*).

As its name implies, a linear regression assumes a linear relationship, which sometimes does not correspond to the data. By looking at the available data, however, the assumption of linear relationships can be justified. Also the choice of only one independent variable, namely, the year of report, seems to be justified when examining the data; the corresponding graphs can be obtained from the results section.

A critical aspect of this model is the so‐called extrapolation, which is the calculation and interpretation of values outside the range for which data are available for the independent variable. However, the increase in the case numbers over the last decades also tends to imply linear behavior for the upcoming years, thus an estimation prognosis for these years is feasible on the basis of the available data. In the following, this prognosis is referred to as “trend.”

## 3 RESULTS

### 3.1 Laser procedures in total

The analyses in this section show the extent to which the use of laser procedures in German hospitals has changed over the years under review.

**Figure 1:**
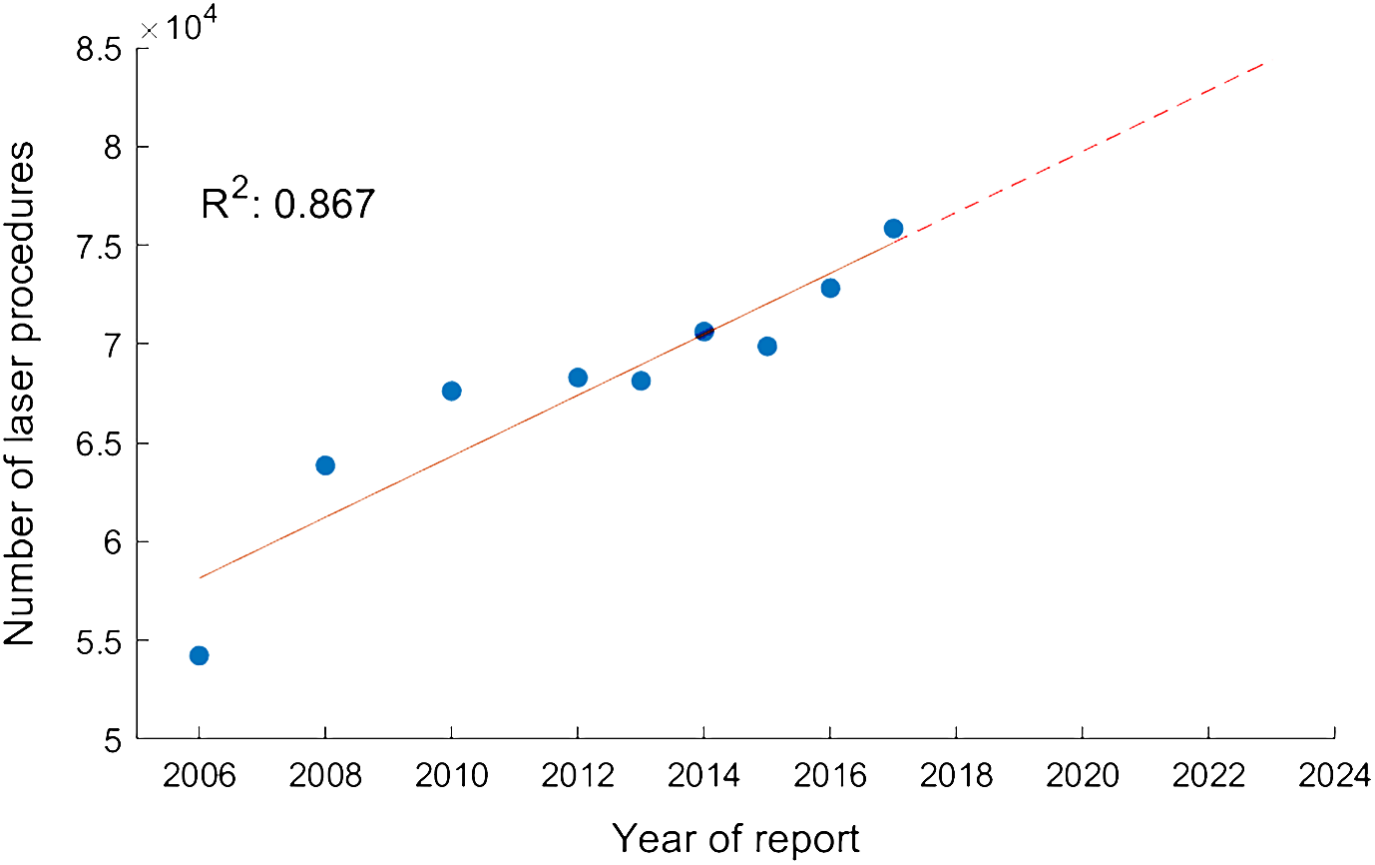
Total number of laser procedures. The trend is indicated by the dashed line. Exact values can be found in the Appendix

Intercept and slope of the calculated linear regression can be deduced from the following equation. This linear equation is shown in red in Figure 1, where the corresponding coefficient of determination *R* ^2^ can also be found.

$$Y=-3.0373\cdot 10^{6}+1,543.1\cdot x_{1}$$

By relating the number of laser procedures performed each year to the number of hospitals that performed laser procedures in the corresponding year of report, Table 2 results, showing the average number of laser procedures annually conducted at a hospital with appropriate laser equipment.

**Table 2:** Average number of laser procedures conducted at a hospital that was performing laser procedures in the specific year of report

Year of report	Laser procedures per hospital (rounded)
2006	149
2008	148
2010	154
2012	140
2013	139
2014	142
2015	139
2016	152
2017	149

### 3.2 Body regions and coagulation in detail

**Figure 2:**
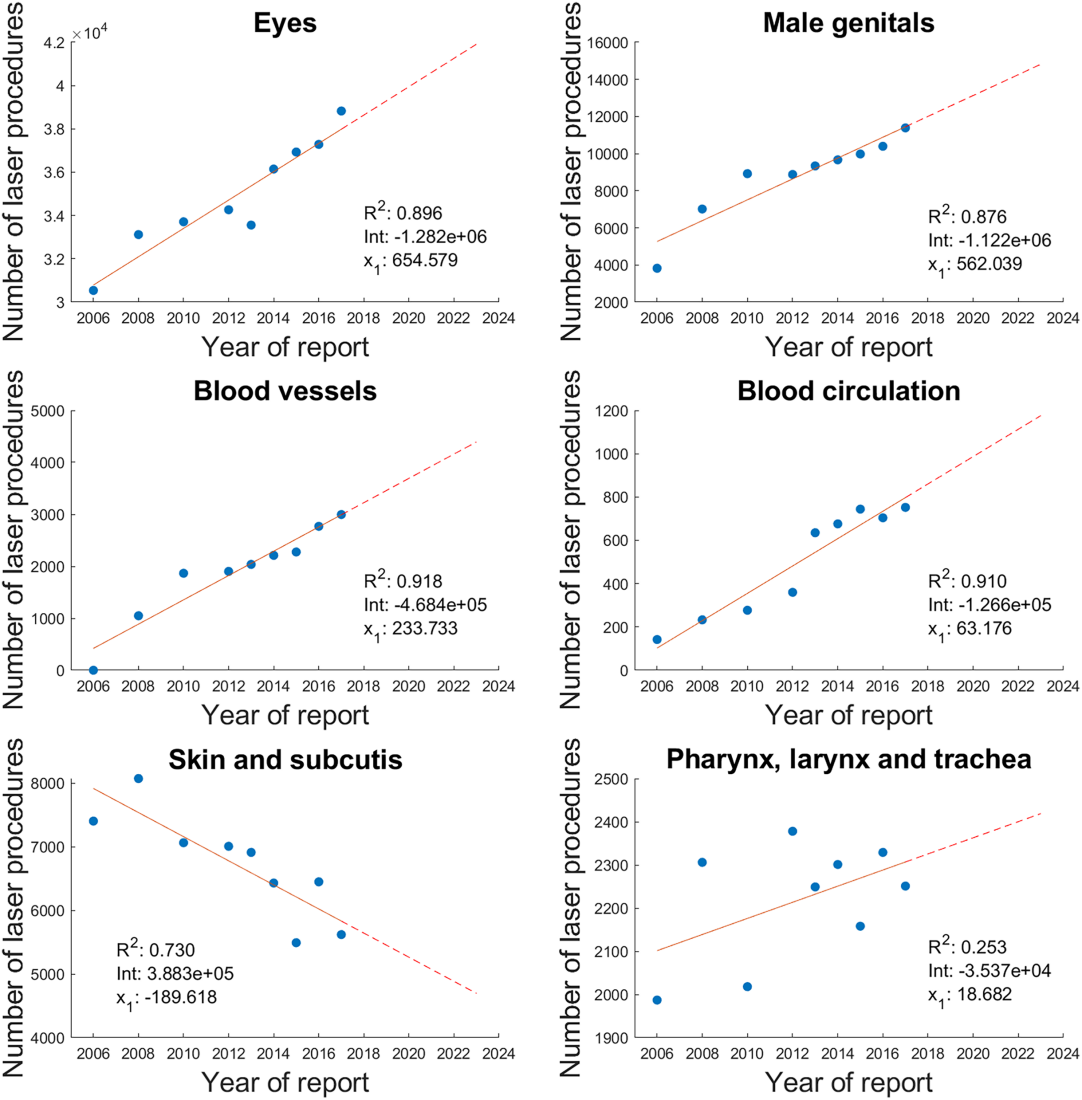
Number of laser procedures carried out in the specific body regions. For each diagram the coefficient of determination *R* ^2^ as well as the values for the intercept *Int* and the slope *x*_1_ of the predictor “Year of report” are given. The respective trend is indicated by the dashed line in each case. Exact values can be found in the Appendix

**Figure 3:**
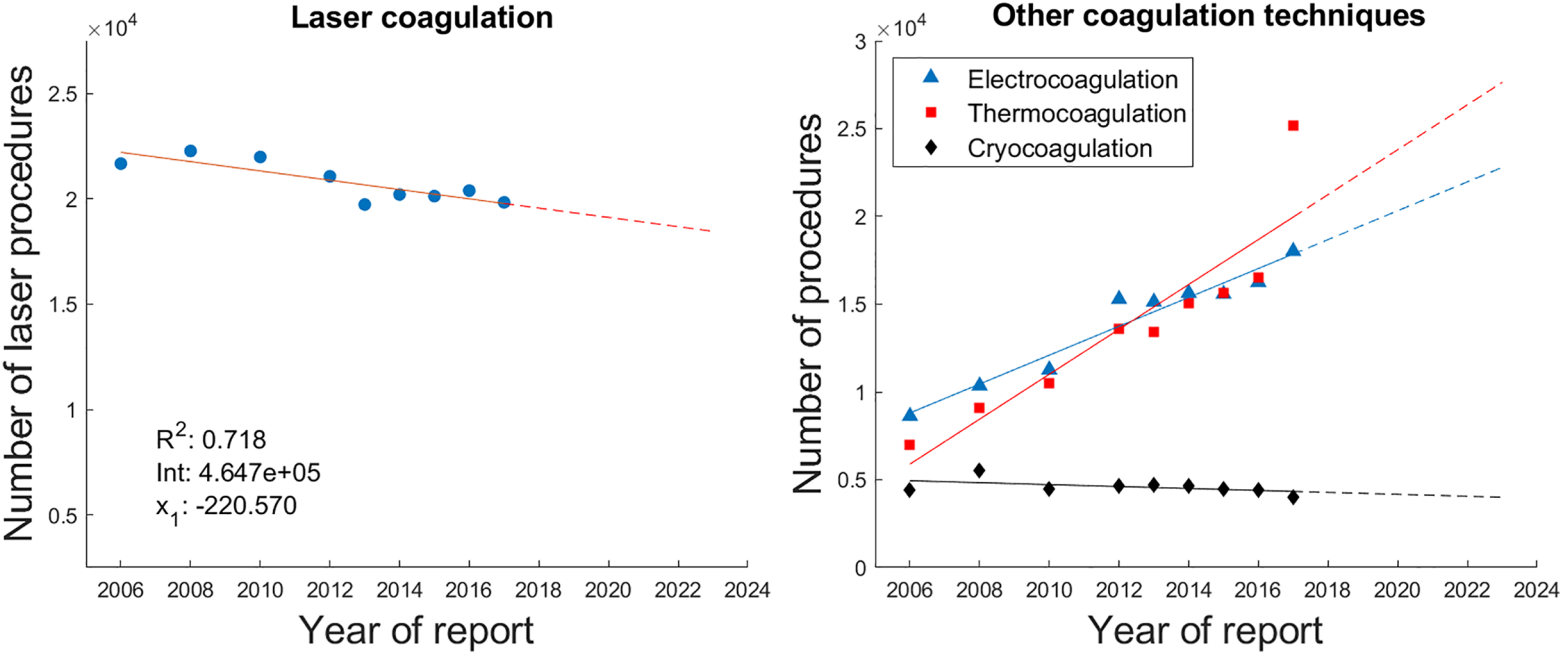
Left: Case numbers of the laser coagulation with the coefficient of determination *R* ^2^ and the values for the intercept *Int* and the slope *x*_1_ of the predictor “Year of report.” Right: Case numbers of three other coagulation techniques (electrocoagulation: *R* ^2^ = .943, *Int* = −1.642e+06, *x*_1_ = 822.879; thermocoagulation: *R* ^2^ = .817, *Int* = −2.560e+06, *x*_1_ = 1279.291; cryocoagulation: *R* ^2^ = .243, *Int* = 1.158e+05, *x*_1_ = −55.236). Note: A case number can account for both one of the coagulation techniques and one of the previously considered six body regions simultaneously. The respective trend is indicated by the dashed line in each case. Exact values can be found in the Appendix

This section of the results chapter separately focuses on some body regions in which the medical laser is used. For this purpose, those regions with the highest number of cases were selected. In addition, the laser coagulation method is regarded, as it plays an important role across all medical disciplines and therefore has a correspondingly high number of cases. Figures 2 and 3 come up with details, particularly also on the calculated linear regressions.

The procedure most prominent in the body region of the eye is laser retinopexy with approximately half of all annual eye cases. With about 12 000 cases per year the removal of diseased retinal and choroidal tissue by laser is the second most frequent treatment in this region. Concerning the male genitals, the transurethral excision and removal of prostate tissue by laser dominates the case numbers: All cases shown in the corresponding graph in Figure 2 are attributable to these procedures. In the body region of the blood vessels, all case numbers are related to the prevention, excision and stripping of varicose veins by laser.

In the field of blood circulation, the cases are a result of both the nonsurgical therapeutic intervention of laser angioplasty and laser ablation with endovascular endoscopic control for ablative interventions in tachyarrhythmia. Over the course of all years, case numbers in laser angioplasty have been reported mainly in the area of the vessels of the lower legs, the femoral arteries and the coronary artery. Concerning the field of pharynx, larynx and trachea, the partial laryngectomy by endoscopic laser resection is the major determinant for case numbers.

A distinction must be made between two dominant procedures in the area of skin and subcutis: On the one hand, the removal of superficial skin layers by laser therapy is considered. Especially skin areas on the head, chest wall and back are treated here. The number of these procedures remained stable over the years of report at around 2000 cases per year. The second laser application in this field is the removal of diseased tissue on the skin and subcutis, which is more prominent than the first method mentioned and which is responsible for the trend shown in Figure 2. The predominant body regions here are head, chest wall, back, hand and foot, as well as the groin and genital regions.

According to the data of the quality reports, no laser was used for procedures in the following body regions in any of the years of report: nervous system, endocrine glands, ears, lungs and bronchus (for this body region there are around 100 counts for PDT per year), jaw and cranial bone, mamma.

Laser coagulation is a procedure that is used in many of the medical disciplines reported on, predominantly for the removal of retinal and choroidal tissue, operations on the nasal concha and the removal of diseased tissue of the vulva. In order to relate laser coagulation in terms of its frequency of application to other coagulation techniques listed in the quality report, Figure 3 also illustrates the case numbers of electrocoagulation (destruction of tissue by means of high‐frequency currents), thermocoagulation (destruction of tissue by exposure to high temperatures) and cryocoagulation (destruction of tissue by icing). Infrared coagulation (destruction of tissue by absorption of infrared radiation) has negligible case numbers and is therefore not depicted. The rather new technique of laser thermocoagulation does not have any equivalent in the quality reports. The case numbers that arise from this technique could therefore be assigned either to laser coagulation or to thermocoagulation.

### 3.3 Types of lasers

**Figure 4:**
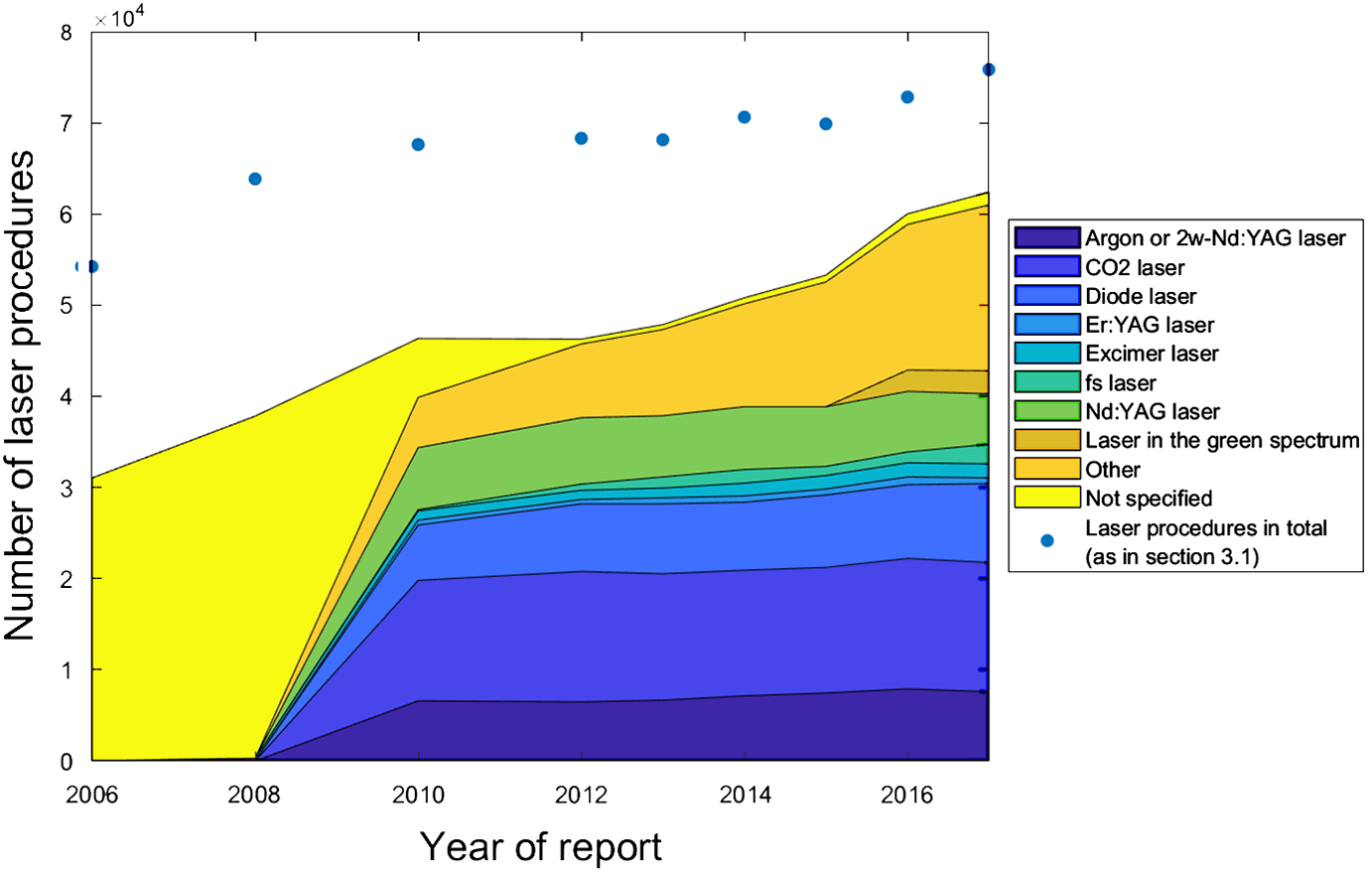
Different types of lasers used in the German hospitals. Due to the structure of the quality reports, apart from the Excimer laser no subdivision of the lasers used was possible for the years 2006 and 2008, respectively. For the category “not specified,” the Operationen‐ und Prozedurenschlüssel (OPS) codes 5‐985 and 5‐985.y have been merged as both codes represent unspecified categories. Exact values can be found in the Appendix

Furthermore, it was investigated which type of laser is used to which extent in the hospitals. Figure 4 shows these data over the years of report. It should be noted in this regard that no subdivision of the lasers used (apart from the Excimer laser) was made in the quality reports of the years 2006 and 2008, which is correspondingly reflected in the figure. Although these years do not contain information regarding laser types, they nevertheless contribute to the trend depicted. Therefore, they were not excluded from the analysis.

According to the logics of the quality reports, each time a laser type is referred to, the code for the procedure in which this laser is used must also be mentioned. However, this is not the case vice versa: a procedure can also be coded without additional information, which means that the total number of procedures mentioned in Section 3.1 (for an easier comparison also displayed in Figure 4) is higher than the total number of procedures reported in this section.

## 4 DISCUSSION

Although it must be noted that the figures reported in the present publication do not include the outpatient medical sector, the trend over the 9 years of the report clearly points in the direction that the use of laser‐based methods and procedures in German hospitals is increasing with regard to the number of cases. As the only predictor in the calculated linear regression, the years of report can explain 86.7% of the variance of the case numbers, which corresponds to a good model adjustment. In line with this model, it can therefore be assumed that the number of uses of medical lasers will continue to rise in the upcoming years. On the basis of the regression equation reported, the corresponding case numbers can be predicted as desired. However, one has to bear in mind that—as always—only a forecast for a couple of years might be reasonable.

Taking account of the two different statistics presented on the numbers of hospitals, two opposing trends are noticeable: On the one hand, the number of available hospitals in Germany decreased continuously over the period examined (reflecting the general economic situation of German hospitals and the health policy of merging and closing hospitals 35), while on the other hand, the number of hospitals that carry out laser procedures increased steadily. In addition to the general increase in the case numbers of laser procedures, this trend is a further indication that laser procedures are of growing importance for hospitals.

Because both case numbers and the number of clinics carrying out laser procedures are increasing, the quotient of these two figures remains relatively stable. This should, however, not be interpreted as stagnation, but rather as balanced growth of both figures. On average, each of these clinics performs around 150 laser procedures per year, that is, about three per week.

Looking at the observations in Section 3.2, it becomes clear that this general trend cannot be transferred to every region of the body where a laser can potentially be used medically: While the regions eyes, male genitals, blood vessels and blood circulation are in line with the overall trend, skin and subcutis show declining case numbers. Again, the years of report as the only predictor in the calculated linear regressions can each explain an appropriate amount of the variance of the case numbers, indicating a good model adjustment in each of the five aforementioned analyses. No trend is discernible for the Pharynx, larynx and trachea body region.

In Section 3, the procedures that contribute mainly to the case numbers in the different separately assessed application regions of the laser were mentioned. It is advisable to include these procedures in the discussion of the trends presented. Doing this by consulting PubMed, it is noticeable that those procedures for which an increase in the number of cases can be reported also an increase in the number of publications on the specific topic can be observed 36: The regions eyes (Search details: laser and retinopexy, laser and eye or opthalmology), male genitals (laser and prostate) and blood vessels (laser and varicose veins) show this behavior.

For the area of blood circulation (laser and angioplasty, laser and tachyarrhythmia), by contrast, this relation cannot be observed 36. The trend in the case numbers shown above is therefore not substantiated by a corresponding trend in the publication statistics. Since this body region has by far the lowest case numbers, it is possible that a pattern like this is strongly influenced by a few individual hospitals. A look at the data set of this study could confirm this assumption: A total of 86 clinics used a laser procedure in the field of blood circulation at least in one of the years of report, but most of them at a level of less than 20 annual cases; only five clinics exceeded the mark of 65 annual cases in one or more years of report, all in the year of report 2013 or later. The use of a laser in the area of laser angioplasty and laser ablation with endovascular endoscopic control for ablative interventions in tachyarrhythmia is thus dominated by a few clinics. Nevertheless, the trend shown is existing and the two procedures mentioned could have increasing numbers of applications in further hospitals in the upcoming years.

Checking PubMed for the relevant terms for skin and subcutis (laser and skin and removal, laser and skin and destruction) did in fact not show any decreasing publication statistics 36, even though the increase is far less pronounced than in the procedures of the previous paragraphs. Since around the year 1999, the number of publications has virtually stagnated. This indicates that during the years of report hardly any new groundbreaking examination and/or treatment methods have been added in this area. Existing methods, meanwhile, may have migrated from the sector of clinical care, which is considered in the present study, to the outpatient medical sector; in particular, the area of esthetic surgery, on which the quality reports do not provide any specific information, could be a precursor in this regard. For the case numbers at German hospitals, the observed trend results then for this body region.

As mentioned in Section 3, the falling case numbers in the area of Skin and subcutis are particularly attributable to the area of laser removal of diseased tissue. It was examined whether other removal methods had increased quantitatively in the same period. This was not true for any of the procedures, namely, electrocaustics, electrochemotherapy, cryosurgery and infrared coagulation. This shows that there has been no shift from laser‐based to nonlaser‐based methods, but rather supports the hypothesis of the previous paragraph that established methods have migrated to the outpatient medical sector while at the same time lacking innovations.

The last body region considered was pharynx, larynx and trachea. The very low coefficient of determination of about 0.25 already indicates an insufficient model adaptation, so the observed trend is rather fragile. It is considered useful to wait for several future quality reports and then make reliable statements with an extended data set. A further interpretation of the case numbers of this body region will therefore not be given at this point.

For the case numbers in Figure 1 as well as for those of the areas “eyes,” “male genitals,” “blood vessels,” and “blood circulation” in Figure 2, the following pattern can be seen: For the years 2006 to 2010, there is an increase in the respective case numbers, followed by a stabilization/saturation, again followed by a further increase from the year 2014 on. This pattern is consistent with the healthcare business cycle that approaches a sinusoidal cycle of 10 to 20 years (depending on technology as well as accepted and approved medical innovations) 37, 38. In the abovementioned figures, the case numbers snake around the linear regression curve. Thus, the linear regression can be interpreted as a long‐term trend of the sinusoidal behavior.

Figure 3 indicates that laser coagulation has a decreasing number of cases throughout the years under review. In order to determine whether this is a method specific trend or a trend affecting the coagulation technique in general, the case numbers of electrocoagulation, thermocoagulation and cryocoagulation were as well analyzed. While the numbers of the latter stagnate, the other two coagulation methods can obtain steady increases. The overall pattern thus reveals that laser coagulation seems to be constantly being replaced by electrocoagulation and thermocoagulation, respectively. This is in line with a study that attributes a better outcome to these two procedures than to laser coagulation 39. The already mentioned imprecise assignment of case numbers regarding the laser thermocoagulation technique should not or only marginally influence this pattern, since this technique is still very novel 40.

The last aspect considered in the results section of this study was the different types of lasers used in the clinics. The results show that the CO_2_ laser is the most commonly used laser in German hospitals, which is in line with the literature 31, 41. Diode lasers and the Nd:YAG laser also play an important role in the application statistics, as do frequency‐doubled Nd:YAG lasers and argon lasers. Due to the long‐term history and clinical experience, the relative importance of CO_2_ and Nd:YAG laser was to be expected.

Unfortunately, the quality reports do not provide any information about what type of laser is preferably used in which medical discipline. In particular, this makes it unfeasible to assess why besides argon and frequency‐doubled Nd:YAG lasers the category of “lasers in the green spectrum” coexists. It is open to discussion whether one reason for this subdivision may be the fact that since about the year 2016 expenses for prostate vaporization with a so‐called “green light laser” are reimbursed by some German health insurances. Thus, this subdivision may have grown historically; in this case, the figures for the two categories mentioned can be added together.

All in all, the results reveal stable application quantities of the different laser types mentioned; no laser stands out showing extreme increases or decreases in the number of cases. Despite the increasing number of applications of medical laser systems shown in the present paper, hospitals are still investing cautiously in new laser equipment. In fact, the figures remained extremely stable, which indicates that laser systems already installed appear to be regularly replaced by new systems at the end of their lifetime, but that investments in an expansion of the machine pool are still negligible. Considering the cumulative figures for all laser types, the already discussed increase in the case numbers of laser‐based medical procedures becomes visible once again.

## 5 CONCLUSION

For the first time, the present study can quantitatively substantiate the importance of the medical laser in everyday clinical practice. Based on the reported trend, it can be assumed that this figure will rise considerably in the future. However, a detailed look at different body regions and coagulation techniques in which the medical laser is used did not reveal a uniform picture.

The investigation of the laser types used confirmed the importance of CO_2_ lasers for medical applications, as did diode lasers and lasers based on doped YAG crystals, together with argon lasers. Interestingly, none of the lasers showed a significant increase or decrease in the case numbers throughout the years of report.

The figures presented and the analyses collected on this basis relate to the sector of German hospitals. The outpatient medical sector and the situation in other countries are therefore not primarily covered. However, it is assumed that comparable trends may be reflected there.

## AUTHOR CONTRIBUTIONS

M.S. obtained the data and wrote the MATLAB script to evaluate the data. He did the data evaluation and interpretation. He prepared the manuscript and drafted it. M.H. and B.L. helped to establish the statistical analyses. Dr‐Ing F.K., Prof Dr F.S. and Prof Dr‐Ing. M.S. guided the general research strategy and gave a critical revision of this manuscript. All authors read and approved this manuscript.

## CONFLICT OF INTEREST

The authors declare that there are no competing financial or nonfinancial interests in relation to the work described.

## Appendix 1

### OPS Codes of the analyzed laser procedures

The following 240 laser procedures were included in the analysis: 3‐302, 5‐091.41, 5‐097.4, 5‐112.01, 5‐121.1, 5‐123.01, 5‐126.6, 5‐131.5, 5‐133.3, 5‐133.4, 5‐133.6, 5‐133.8, 5‐133.80, 5‐135.31, 5‐135.41, 5‐135.51, 5‐136.1, 5‐137.0, 5‐142.0, 5‐142.2, 5‐154.2, 5‐155.3, 5‐155.4, 5‐155.7, 5‐159.3, 5‐159.30, 5‐159.31, 5‐159.32, 5‐159.33, 5‐159.34, 5‐159.35, 5‐159.36, 5‐159.3x, 5‐159.3y, 5‐210.3, 5‐215.02, 5‐250.31, 5‐272.61, 5‐273.91, 5‐289.01, 5‐292.31, 5‐300.31, 5‐302.5, 5‐363.6, 5‐371.36, 5‐371.46, 5‐371.56, 5‐378.a0, 5‐385.a, 5‐385.a0, 5‐385.a1, 5‐419.4, 5‐422.31, 5‐422.41, 5‐422.51, 5‐422.x1, 5‐433.31, 5‐433.41, 5‐433.51, 5‐433.x1, 5‐451.41, 5‐451.51, 5‐451.61, 5‐451.91, 5‐451.a1, 5‐451.x1, 5‐452.31, 5‐452.41, 5‐452.51, 5‐452.81, 5‐452.91, 5‐452.x1, 5‐482.4, 5‐482.40, 5‐482.41, 5‐482.42, 5‐482.4x, 5‐482.4y, 5‐501.6, 5‐501.60, 5‐501.61, 5‐501.62, 5‐501.63, 5‐501.6x, 5‐513.24, 5‐513.25, 5‐526.24, 5‐526.25, 5‐585.2, 5‐601.4, 5‐601.40, 5‐601.41, 5‐601.42, 5‐601.4x, 5‐601.4y, 5‐601.7, 5‐601.70, 5‐601.71, 5‐601.7x, 5‐671.00, 5‐671.10, 5‐672.12, 5‐681.51, 5‐681.61, 5‐702.32, 5‐712.12, 5‐754.4, 5‐789.5, 5‐913.4, 5‐913.40, 5‐913.44, 5‐913.45, 5‐913.46, 5‐913.47, 5‐913.48, 5‐913.49, 5‐913.4a, 5‐913.4b, 5‐913.4c, 5‐913.4d, 5‐913.4e, 5‐913.4f, 5‐913.4g, 5‐913.4x, 5‐913.4y, 5‐913.a, 5‐913.a0, 5‐913.a4, 5‐913.a5, 5‐913.a6, 5‐913.a7, 5‐913.a8, 5‐913.a9, 5‐913.aa, 5‐913.ab, 5‐913.ac, 5‐913.ad, 5‐913.ae, 5‐913.af, 5‐913.ag, 5‐913.ax, 5‐913.ay, 5‐915.1, 5‐915.10, 5‐915.14, 5‐915.15, 5‐915.16, 5‐915.17, 5‐915.18, 5‐915.19, 5‐915.1a, 5‐915.1b, 5‐915.1c, 5‐915.1d, 5‐915.1e, 5‐915.1f, 5‐915.1g, 5‐915.1x, 5‐915.1y, 5‐915.5, 5‐915.50, 5‐915.54, 5‐915.55, 5‐915.56, 5‐915.57, 5‐915.58, 5‐915.59, 5‐915.5a, 5‐915.5b, 5‐915.5c, 5‐915.5d, 5‐915.5e, 5‐915.5f, 5‐915.5g, 5‐915.5x, 5‐915.5y, 5‐921.4, 5‐921.40, 5‐921.41, 5‐921.42, 5‐921.43, 5‐921.44, 5‐921.45, 5‐921.46, 5‐921.47, 5‐921.48, 5‐921.49, 5‐921.4a, 5‐921.4b, 5‐921.4c, 5‐921.4d, 5‐921.4e, 5‐921.4f, 5‐921.4g, 5‐921.4h, 5‐921.4j, 5‐921.4k, 5‐921.4m, 5‐921.4x, 5‐921.4y, 5‐985, 5‐985.0, 5‐985.1, 5‐985.2, 5‐985.3, 5‐985.4, 5‐985.5, 5‐985.6, 5‐985.7, 5‐985.x, 5‐985.y, 8‐835.e, 8‐836.2, 8‐836.20, 8‐836.21, 8‐836.22, 8‐836.23, 8‐836.24, 8‐836.25, 8‐836.26, 8‐836.27, 8‐836.28, 8‐836.29, 8‐836.2a, 8‐836.2b, 8‐836.2c, 8‐836.2d, 8‐836.2e, 8‐836.2f, 8‐836.2g, 8‐836.2h, 8‐836.2j, 8‐836.2k, 8‐836.2m, 8‐836.2x, 8‐836.2y, 8‐837.1, 8‐837.10, 8‐837.11, 8‐837.1y, 8‐837.e, 8‐838.2, 8‐838.20, 8‐838.21, 8‐838.22, 8‐838.23, 8‐838.24, 8‐838.25, 8‐838.2x, 8‐838.2y

### OPS Codes of the analyzed laser types

The following 11 laser types were included in the analysis: 5‐985, 5‐985.0, 5‐985.1, 5‐985.2, 5‐985.3, 5‐985.4, 5‐985.5, 5‐985.6, 5‐985.7, 5‐985.x, 5‐985.y

**Table:** **TABLE A1** Data underlying the figures presented in this publication

Year	2006	2008	2010	2012	2013	2014	2015	2016	2017
Number of hospitals	1940	1922	1872	1737	1746	1757	1742	1722	1715
Number of hospitals with LP	364	432	440	489	491	499	504	480	509
LP in total	54 232	63 852	67 620	68 302	68 135	70 626	69 885	72 826	75 842
LP per hospital^a^	149	148	154	140	139	142	139	152	149
Eyes	30 538	33 119	33 709	34 266	33 558	36 146	36 933	37 285	38 826
Male genitals	3823	7018	8922	8880	9340	9668	9982	10 396	11 389
Blood vessels	0	1049	1867	1902	2037	2211	2276	2769	2996
Blood circulation	142	233	277	360	635	676	744	704	752
Skin and subcutis	7405	8074	7065	7009	6913	6431	5492	6450	5620
Phar., lar., trachea	1987	2306	2018	2378	2249	2301	2158	2329	2251
Laser coagulation	21 673	22 275	21 989	21 072	19 733	20 208	20 134	20 391	19 835
Electrocoagulation	8624	10 356	11 268	15 286	15 135	15 633	15 580	16 257	18 021
Thermocoagulation	7017	9122	10 482	13 572	13 417	15 038	15 618	16 517	25 169
Cryocoagulation	4396	5555	4451	4667	4724	4620	4470	4443	4011
Excimer laser	0	289	1010	1006	1073	1392	1474	1545	1534
Green spectrum	0	0	0	0	0	0	0	2337	2527
Argon, 2w‐Nd:YAG	0	0	6592	6479	6664	7141	7449	7904	7588
Nd:YAG laser	0	0	6799	7281	6703	6885	6536	6637	5520
Er:YAG laser	0	0	538	485	665	702	659	858	631
CO_2_ laser	0	0	13 212	14 304	13 872	13 779	13 783	14 310	14 184
Diode laser	0	0	6082	7421	7668	7463	7947	8093	8659
fs laser	0	0	138	678	1218	1495	1002	1191	2153
Other	0	0	5505	8084	9462	11 287	13 694	15 988	18 198
Not specified	31 038	37 535	6458	529	535	662	742	1158	1406
PDT	433	3028	4804	4748	4695	5291	5488	4393	4726

a LP per hospital has been rounded.

Abbreviations: LP, laser procedures; PDT, photodynamic therapy.
